# Prediction of Mental Health Services Use One Year After Regular Referral to Specialized Care Versus Referral to Stepped Collaborative Care

**DOI:** 10.1007/s10597-016-0046-y

**Published:** 2016-07-18

**Authors:** Mirjam van Orden, Stephanie Leone, Judith Haffmans, Philip Spinhoven, Erik Hoencamp

**Affiliations:** 1 Parnassia Groep, Parnassia Academie, Kiwistraat 32, 2552 DH The Hague, The Netherlands; 2 Trimbos Institute, Netherlands Institute of Mental Health and Addiction, Utrecht, The Netherlands; 3 Medical Chronobiology, PsyQ, The Hague, The Netherlands; 4 Faculty of Social and Behavioral Sciences, Leiden University, Leiden, The Netherlands; 5 Indigo Zorgservice, Parnassia Groep, The Hague, The Netherlands

**Keywords:** Collaborative mental health care, Prediction, Mental health services use, Service concept

## Abstract

Referral to collaborative mental health care within the primary care setting is a service concept that has shown to be as effective as direct referral to specialized mental health care for patients with common mental disorders. Additionally it is more efficient in terms of lower mental health services use. This post-hoc analysis examines if treatment intensity during 1-year of follow-up can be predicted prospectively by baseline characteristics. With multilevel multivariate regression analyses baseline characteristics were examined as potential predictors of visit counts. Results showed that only the enabling factors service concept and referral delay for treatment had a significant association with mental health visit counts, when outcome was dichotomized in five or more visits. Inclusion of the outcome variable as a count variable confirmed the predictive value of service concept and referral delay, but added marital status as a significant predictor. Overall, enabling factors (service concept and referral delay) seem to be important and dominant predictors of mental health services use.

## Introduction

As a result of increasing demands and limited resources, mental health care is under increasing pressure to enhance efficiency (Schaefer et al. 2003). For this reason, high quality short-term treatment is more appealing than ever. Stepped care is an approach where the initial treatment is the least restrictive of those available, but still likely to provide significant health gain (Bower and Gilbody 2005). Only for patients who fail to respond, the treatment intensity is ‘stepped up’, i.e. referral to specialized mental health care. This prevents unnecessary treatment for most of the patients who will recover with minimal interventions (Meeuwissen et al. 2008). In recent years, the first treatment step is increasingly offered in the primary care setting, supported by liaison-consultation functions (i.e. collaborative care). Integrating specialized mental health services in primary care was one of the most fundamental recommendations of the World Health Organization in 2001 (WHO 2001). Stepped/collaborative care has been shown to be effective for patients with anxiety, depression and addictive problems with regard to a decrease in complaints (Meeuwissen et al. 2008; van Orden et al. 2009; Woltmann et al. 2012). With regard to efficiency there are indications that collaborative care is associated with decreased mental health care utilization (van Orden et al. 2009) and a subsequent decrease in costs (Drummond et al. 2009), even on the longer term (van Orden et al. 2015).

An alternative approach to stepped care is matched care. In this approach the patient is allocated to the most appropriate therapy based on available intake information. As a result, treatment method, treatment intensity and treatment setting may vary (van Straten et al. 2006). Quick and appropriate allocation could enhance continuity and efficiency of care. Crucial in this approach is to integrate all relevant criteria in the allocation process that potentially predicts response to the treatment. This approach poses a major problem, however, as factors which are stable predictors for subsequent treatment intensity have to be known and the available empirical evidence on such predictors is inconclusive.

An important and widely used theoretical prediction model is Andersen’s model of health services use (Andersen 1995). According to this model, people’s use of health care is a function of (a) their predisposition to use services, (b) need for care, and (c) enabling factors. The predisposition to use services includes demographic factors, like age and gender, social structures and health beliefs. Social structure is measured by various factors that determine the status of a person in the community, his or her ability to cope with presenting problems, and the state of health of the physical environment. Social structures include factors such as education, occupation, ethnicity, culture, social networks and social interactions. Health beliefs are attitudes, values, and knowledge about health and health services that might influence perceptions of need and use of health services.

The subjective evaluation or perceived need for care is based on the perception of one’s own general health and functional state, as well as how symptoms of illness, pain, and worries about health are experienced. Furthermore, the judgment whether problems are of sufficient importance and magnitude to seek professional help is an important factor in subjective evaluation. Objective or evaluated need for care represents professional judgment about people’s health status and need for care.

Regarding enabling resources, two types of resources must be present for health care use to take place: community and personal enabling resources. Therefore, health personnel and facilities must be available. On a personal level people must have the means and know-how to approach these services and make use of them. Income, health insurance, a regular source of care, and travel and waiting times are relevant variables in this context. One concern about Andersen’s (Andersen 1995) initial model was that organizational factors were not given enough attention. Knowledge about the organization of care (i.e. the various kinds of medical care providers and types of health services organizations in the community) was expected to improve the ability to explain and predict care. Accordingly, Andersen suggests including organizational measures as additional enabling factors.

The aim of the randomised controlled trial assessing the effectiveness and efficiency of a Dutch collaborative care program for treatment of common mental disorders was to examine the effectiveness, efficiency, accessibility and acceptance of CCP compared to usual specialized mental health care. The results of this trial confirmed our hypothesis that that for the treatment of common mental disorders referral to collaborative care was (at least) equally effective as direct referral to specialized mental health care, but resulted in significantly lower mental health services use, even 5 years after referral (van Orden et al. 2015). In this study however, 32 % of the patients who were initially treated within the collaborative care condition still needed subsequent referral to specialized mental health services. This raises the question whether the investigated stepped care approach is suitable for all patients with common mental disorders or whether there is an identifiable group of patients who do not benefit sufficiently from the collaborative care program and for whom it would be better to be referred directly to specialized care/matched care? To answer this question we performed a post-hoc analysis of the data of the above mentioned RCT to find out whether treatment intensity was dependent on dispositional, need for care and enabling factors, as described by Andersen (1995). In other words: is it possible to identify a subgroup of patients for whom matched care would be more appropriate than collaborative care?

While the primary care sector is the fastest growing sector of mental health care delivery, only a small number of studies have examined correlates of mental health care use in primary care (Lindsay Nour 2009). Lindsay et al. found that need for care variables, including mood disorders, anxiety and substance use disorders seem to be most strongly related to treatment use compared to predisposing and enabling factors. In a study in the Netherlands, Schaefer et al. (2003) investigated the impact of patient characteristics on the decision and referral process to brief (maximum of six visits) and longer-term treatment models by clinicians. They concluded that patients who have assets and a number of aspects in their lives in which they function well (especially good relationships) are considered to be better candidates for brief-term treatment then patients who have besides their target problem also other weaknesses and problems in living. In terms of Andersen’s model, certain predispositional and need for care factors have an influence on the allocation process of clinicians to brief or longer-term treatment.

The primary aim of the present study was to conduct a post-hoc analysis to identify predispositional, enabling and need for care factors that predict mental health services use within the year after referral to either a stepped collaborative care program or specialized mental health services.

## Methods

### Setting

This post-hoc analysis uses data from a previously performed randomized controlled trial (van Orden et al. 2009). For this trial, general practitioner (GP) practices (*n* = 27) were randomized to one of two conditions: either the practice continued its usual way of referring patients to specialized mental health services if indicated (care as usual, CAU; *n* = 63 patients) or the GP practice referred patients to an on-site mental health professional who could see the patient for a maximum of five sessions with subsequent referral to specialized mental health care if indicated (stepped collaborative care in the primary care setting, CCP; *n* = 102 patients). The general agreement between the CCP professional and the GP was that the GP would refer all patients to the CCP professional initially, unless there were patient-related reasons for directly referring a patient to specialized mental health care (such as patients needing acute mental health care). The regular assessment of GPs’ motivation for referral to either CCP or CAU was not part of the study protocol. In the CCP condition, a brief time-limited intervention with a maximum of five sessions is offered in a period of 6 months, based on a time-limited intervention model with a cognitive behavioural approach (Rijnders et al. 2002) by community psychiatric nurses or psychologists. If indicated by the mental health professional, consultation of the patient by a psychiatrist was possible. The mental health professional had regular face-to-face contacts with the GP. A team of psychiatrists met the mental health professional once a month and conducted regular meetings with the GP. The CAU condition encompassed regular referral by the GP practices to specialized mental health care.

In the trial no significant baseline differences (sociodemographic characteristics, diagnosis, severity of psychopathology) were found between the patients that were referred to either the CCP condition or the care as usual condition (van Orden et al. 2009). Moreover, at 1-year follow-up there were no significant differences between the two conditions with respect to severity of psychopathology, quality of life and patient satisfaction. Collaborative care did lead to significantly higher levels of GP’s satisfaction and proved to be more efficient than care as usual with respect to referral delay, defined as the waiting time for patients between referral by GP and first face-to-face appointment with a mental health professional, duration of treatment, number of appointments and related treatment costs.

The retrospective study was performed between January and September 2013.

Written informed consent and the approval of the accredited Medical Research Ethics Committee (METiGG) was obtained.

### Measurements

Based on the variables collected within the RCT we were able to integrate a number of factors, assessed at baseline, that potentially predict people’s use of health care according to Andersen’s prediction model (Andersen 1995). Regarding to predispositional factors, participating patients were queried about gender, age, education and marital status. As objective need measures we used the Mini International Neuropsychiatric Interview (Sheehan et al. 1998), the Clinical Global Impression Scale (CGI) (Guy 1976) and an assessment of symptom duration. The Mini International Neuropsychiatric Interview was conducted by trained interviewers for Axis-I diagnosis according to DSM-IV criteria. The GP rated the severity of the patient’s symptoms on a seven-point scale with the CGI and symptom duration on a three-point scale: <3 months, 3–9 months and >9 months. As subjective need factors, the Symptom Checklist (SCL-90) (Derogatis et al. 1973) was used for self-reported mental health problems (Arrindell and Ettema 1986) and the World Health Organization Quality of Life Questionnaire (WHOQOL-Bref) (Group 1998) for self-rated overall quality of life and general health.

As enabling factors we were able to include referral delay which was defined as the waiting time in weeks for patients between referral by their GP and their first face-to-face appointment with a mental health professional and the actual service concept, consisting of the two treatment conditions [i.e., care as usual (CAU) and stepped collaborative care in the primary care setting (CCP)].

### Analysis

The intraclass correlation coefficient (ICC) was calculated to test the relatedness of patients belonging to the same GP (clusters).

For the primary post-hoc analysis, patients were divided into two outcome groups regardless of the original referral condition: patients who finished treatment with five or less visits (‘low service group’) to the mental health care professional and patients who were treated with more than five visits (‘high service group’). The choice for the split on five appointments was based on the service concept of the CCP by offering a maximum of five sessions to a patient with further referral to specialized care, only in case the patient was not sufficiently recovered. We compared baseline predictor variables and drop-out rates (premature termination of treatment) between the high and low service use groups using Chi square tests, non-parametric tests and t-tests.

While only 10 (6 %) of the enrolled patients in the RCT dropped out of the study during the 1-year follow-up period (see van Orden et al. 2009 for details), for 39 % of patients one or more variables were missing in their dataset. To circumvent reduced representativeness of the sample, we conducted multiple imputation (MI) by Markov Chain Monte Carlo (MCMC) simulation with 50 iterations. With a two-level (patient as the first and GP as the second level) multivariate logistic model with maximum-likelihood estimation (MLE), we investigated the associations between the predictors and the dichotomous outcome variable.

Because of the limited statistical power of reducing service use to a dichotomous variable, we carried out a second post-hoc analysis. In a two-level multivariate regression analysis with MLE, we investigated the association of the predictors with the outcome ‘count’ variable (total number of mental health care visits during the one year after referral). Because of the nonnormality of our count data distribution (skewness 2.721; kurtosis 9.245), we log-transformed (ten-log) the outcome count data before conducting the regression analysis.

Statistical analyses of baseline data were performed using SPSS 20 and multilevel regression analyses using Mplus 6 (Muthén and Muthén 1998–2010).

All authors certify responsibility. There are no known conflicts of interest.

## Results

For 159 of the 165 patients who participated in the RCT we were able to obtain data about their mental health services use during the 1 year after enrolment in the RCT. A low mental health services use (five or less visits) was found for 85 patients (60 % CCP, 43 % CAU) and 74 patients had a high mental health services use (more than five visits; 40 % CCP, 57 % CAU).

Within the CCP group, 32 % (*n* = 27) were subsequently referred to specialized mental health care. Of the 68 % (*n* = 58) of the CCP group that were not referred to specialized care, 90 % (*n* = 52) had low mental health services use compared to 10 % (*n* = 8) that had high mental health services use. There was no significant difference in drop-out rates, defined as premature termination of treatment by the patient, between the CCP group (*n* = 9; 8,8 %; for ten patients data about drop-out was missing) compared to the CAU group (*n* = 12; 19 %; for 6 patients data about drop-out was missing).

Table 1 shows the descriptives of all baseline predictor variables of the dichotomized outcome groups. There were no statistically significant differences between the low mental health services use group and the high mental health services use group on any of the baseline variables.

**Table 1:** Demographic and symptom characteristics of patients by mental health services use

	Low use (<5 appointments; N = 85)		High use (≥5 appointments; N = 74)	
	N	%	N	%
*Predispositional factors*				
Age (M ± SD)	40.2 ± 13.3		40.8 ± 13.8	
Gender				
Male	25	29	25	34
Female	60	71	49	66
Marital status				
Not married	45	53	46	62
Married/living together	40	47	28	38
Education^a^				
Primary	9	13	8	13
Secondary	18	27	18	30
Low vocational	20	29	18	30
High vocational	16	24	12	20
University	5	7	5	8
*Need factors*				
Clinical global impression: symptom severity^a^				
Normal, not at all ill	8	10	16	25
Borderline mentally ill	11	14	5	8
Mildly ill	28	35	17	26
Moderately ill	13	17	12	19
Markedly ill	17	22	11	17
Severely ill	2	3	4	6
Extremely ill	–	–	–	–
Symptom duration^a^				
<3 months	23	29	17	24
3–9 months	22	28	18	25
>9 months	35	44	37	51
Diagnosis				
Mood disorder	23	27	26	35
Anxiety disorder	31	37	30	41
Other axis 1 disorder	31	37	18	24
SCL-90 Psychopathology score (M ± SD)^a^	179 ± 61.7		190 ± 62.0	
WHOQOL-bref general evaluative facet (M ± SD)^a^	3.1 ± 0.8		2.9 ± 0.8	
*Enabling factors*				
Treatment delay in weeks (M ± SD)^a^	4.6 ± 8.2		3.6 ± 5.1	

^a^Not all data were available for all persons

The intra-class correlation was 0.044, indicating a very small cluster-effect.

In the multilevel multivariate logistic regression analyses, the enabling factors service concept (OR = 1.261; *P* = 0.012) and referral delay (OR = 0.796; *P* = 0.038) were the only factors with a significant association with the mental health services use, indicating that patients who were referred to the CAU condition had a 26 % Odds of belonging to the high service use group (see Table 2). On the other hand, the longer the waiting time, the lower the ODDS of belonging to the high service group.

**Table 2:** Associations (OR’s and 95 % CI) of predictor variables and outcome variable of the multivariate logistic regression model

	Multivariate logistic regression model (dichotomous outcome; low use = 0)				
	Estimate	SE	OR	95 % CI	Sig
Predispositional factors					
Age	0.06	0.10	1.06	(0.88–1.28)	0.62
Gender (male = 0)	−0.02	0.09	0.98	(0.83–1.17)	0.85
Marital status (not married = 0)	−0.13	0.09	0.88	(0.74–1.04)	0.14
Education (primary school = 0)	0.05	0.10	1.05	(0.86–1.28)	0.62
Need factors					
Clinical global impression: symptom severity (normal = 1)	−0.08	0.10	0.93	(0.76–1.12)	0.43
Symptom duration (<3 months = 0)	0.16	0.09	1.17	(0.98–1.40)	0.09
Diagnosis (mood disorders = 0; anxiety disorders = 1; other axis I disorders = 3)	−0.13	0.11	0.88	(0.71–1.08)	0.21
SCL-90 psychopathology score	−0.04	0.13	0.97	(0.75–1.25)	0.79
WHOQOL-bref general evaluative facet	−0.14	0.15	0.87	(0.66–1.16)	0.34
Enabling factors					
Treatment delay in weeks	−0.23	0.11	0.80	(0.64–0.99)	**0.04**
Service concept (CCP = 0)	0.23	0.09	1.26	(1.05–1.51)	**0.01**

Significant associations at *P* < 0.05 level are shown in bold

The results of the multilevel multivariate Poisson regression model confirmed the significant predictive value of the two enabling factors (service concept *P* = 0.012; referral delay *P* = 0.017), but added marital status as a significant predictor (*P* = 0.003; see Table 3). Married patients are less likely to become a high MHC user compared to non-married patients.

**Table 3:** Associations (OR’s and 95 % CI) of predictor variables and outcome variable of the multivariate Poisson regression model

	Multivariate Poisson regression model (mental health visit counts)			
	Estimate	SE	Estimate/SE	Sig
Predispositional factors				
Age	0.12	0.08	1.39	0.16
Gender (male = 0)	−0.01	0.08	−0.06	0.95
Marital status (not married = 0)	−0.22	0.07	−2.99	**0.00**
Education (primary school = 0)	0.10	0.09	1.10	0.27
Need factors				
Clinical global impression: symptom severity (normal = 1)	−0.03	0.08	−0.40	0.69
Symptom duration (<3 months = 0)	0.13	0.08	1.58	0.11
Diagnosis (mood disorders = 0; anxiety disorders = 1; other axis I disorders = 3)	−0.13	0.09	−1.35	0.18
SCL-90 psychopathology score	−0.04	0.11	−0.33	0.75
WHOQOL-bref general evaluative facet	−0.12	0.12	−1.07	0.28
Enabling factors				
Treatment delay in weeks	−0.21	0.09	−2.39	**0.02**
Service concept (CCP = 0)	0.26	0.11	2.50	**0.01**

Significant associations at *P* < 0.05 level are shown in bold

## Discussion

In this study we examined potential predictors of mental health services use during a 1-year period after referral to either collaborative care in primary care or specialized mental health care. Based on the theoretical concept of the collaborative care model used, we dichotomized mental health services use in high (>5 visits) vs. low (≤5 visits) mental health services use. In the two-level multivariate logistic regression analysis we found that mental health services use was not significantly associated with predispositional or need for care characteristics. In this analysis the enabling factors service concept and referral delay were the sole factors with a significant predictive value on the number of mental health visits patients received. Referral of patients to collaborative care decreases the number of mental health visits compared to direct referral to specialized mental health care. An increase in referral delay decreases the number of mental health visits. In the multivariate regression model (with the outcome variable as a count variable), the predictive value of the enabling factors (service concept and referral delay) was confirmed, but marital status became also a significant predictor with married patients being less likely to receive more mental health visits.

Our findings strongly support Anderson’s conclusions that organizational factors are important enabling factors for mental health care use (Andersen 1995). These factors might even be superior over need and predispositional factors in predicting mental health service use. By finding no evidence that need for care and predispositional factors (except marriage as a potential protective factor) have to be included in the allocation process for referral, we obtained preliminary evidence that stepped care might be preferred over matched care for patients with common mental disorders. Interestingly, the predictive value of marital status is consistent with the findings of Schaefer et al. (Schaefer et al. 2003) that relationship factors (marital status) could have a protective effect on becoming a high mental health services user.

An important feature in which this study differs from most similar studies is the number of visits offered to patients in the primary care setting. Meta-analyses of treatment outcome studies suggest that the largest gains are made during the first six to eight treatment sessions. In Schaefer’s study brief treatment included six visits (Schaefer et al. 2003). In this study, the time-limited treatment model included only five visits, which is less than most short time treatment models.

It also has to be noted that this study has been performed in the Netherlands with a mental health system that differs from mental health systems in other countries, like the U.S. In a review of studies that assessed the use and implementation of Andersen’s model (Babitsch et al. 2012) factors related to the accessibility of mental health care, such as being insured and having a regular source of care have been pointed out as influencers of the likelihood of service use in the U.S. In the Netherlands the above mentioned factors do not have this importance because all Dutch citizens are obliged to buy individual private health insurance with an income dependent contribution. Furthermore each insured person has to register with a single GP, who is assumed to coordinate and preauthorize specialist care (van de Ven and Schut 2008). The evaluation of enabling factors of mental health care use thus has to be performed in the light of the existing health care system. It is possible, that if this study was performed within the U.S., accessibility of mental health care could constitute an important confounder leading to different results. The reform of health insurance in the U.S. following the Dutch universal mandatory health insurance model as described by van de Ven and Schut (2008) could improve enabling factors for U.S. citizens to use mental health care.

This study has some important limitations that have to be addressed in interpreting our results. First, the relatively small sample size limits the power to detect weaker associations between mental health services use and predictor variables and limits the number of potential predictor variables that can be included in the analyses. By recoding our count data into dichotomous categories, we realize that this poses additional problems of loosing information and reducing statistical power even more. Therefore, we additionally conducted a multivariate regression analyses with the outcome variable as a count variable to check whether the findings from our logistic regression analysis could be confirmed.

The second important limitation concerns the rate of missing data. Despite of low levels of attrition, there was a notable rate of missing data, especially data about need for care factors. We assume that the way of collecting these data by sending questionnaires by mail to the patients accounted for the high rate of missing data. However, the availability of an electronic database resulted in a much lower percentage of missing data of actual mental health services use and probably more reliable data on service use than self-reports by the patients themselves. By means of the MI approach, we attempted to increase power and decrease the risk of biased results. The third limitation is partially related to the design of the study, set up to assess the effectiveness and efficiency of collaborative care compared to care as usual. Measurements were chosen that served this primary intent. Therefore, inquiries about relevant aspects related to social structures, like ethnicity, employment, income and health beliefs were limited. Other potential predictors, like self-perceived need of mental health care (Aoun et al. 2004; Sareen et al. 2005) and coping that could also provide valuable information as predictors of mental health services use, could not be included in the analyses. The fourth limitation concerns the follow-up period of 1 year, which could have affected the results of our analyses. 28 % of the collaborative care group and 46 % of the usual care group did not terminate treatment within the 1 year follow-up period (van Orden et al. 2009). Consequently, it is possible that some of the patients, who did not terminate treatment within the follow-up period, could have ended up in the high services group. This is expected to affect especially the care as usual group since nearly half of this group did receive treatment after the 1 year period. This possibility could also be related to the result of the variable referral delay. The longer the referral delay for treatment, the less chance of terminating treatment within the 1-year period. An alternative explanation for the effect of referral delay on mental health visit counts may be the influence of wait time for treatment on patient’s motivation. Gallucci et al. (2005) found that increased wait time for an initial appointment at a community mental health centre adversely affects the rate of kept appointments. The shorter the referral delay, the less likely are missed appointments during treatment. In the context of this study, this would imply that in some cases, low intensity use would not automatically relate to the need for treatment, but to diminished motivation due to long referral delay. A second alternative explanation could be that referral delay, in some cases, could cause a natural decrease in severity of symptoms leading to a decreased need of treatment intensity.

In this study we found no evidence that it is possible to identify a subgroup of patients that will not sufficiently respond to a short-time collaborative care treatment as a first and least intensive step in a stepped care model on the basis of pre-treatment dispositional or need for care factors. The factor that had the largest influence on eventual mental health services use was the organization of care. Paying attention to the support system of the patient could, however, also be of importance for the intensity of the actual treatment process. Further research is needed to, first, investigate the role of quality of relationships as a protecting factor for mental health services use and, second, investigate mental health services use during a longer follow-up period.

Based on our findings and taking into account the discussed limitations of our study, we recommend that in countries where general practitioners play a pivotal role in the referral system as gatekeepers for access to mental health care, it may be appropriate to reconsider referral patterns and offer the first and least intensive mental health treatment step in the primary care setting to all non-acute patients with common mental disorders. This would imply that referral to specialized secondary mental health care should be considered as a second step, offered only to patients, who fail to exhibit sufficient signs of recovery.
